# Knowledge, Attitudes, and Practices of General Physicians towards Mild Cognitive Impairment amidst an evolving era of Interprofessional Collaboration: Insights from a small-scale survey in India

**DOI:** 10.1186/s12875-025-02748-7

**Published:** 2025-02-19

**Authors:** Aarushi Soni, Prajith Carthik, Manoj Kumar Devara, Aysha Rooha, Gagan Bajaj, Sheetal Raj Moolambally, Arun Shirali, Archith Boloor

**Affiliations:** 1 Department of Audiology and Speech Language Pathology, Kasturba Medical College Mangalore, Manipal Academy of Higher Education, Manipal, Karnataka 576104 India; 2 Department of General Medicine, Kasturba Medical College Mangalore, Manipal Academy of Higher Education, Manipal, Karnataka 576104 India

**Keywords:** Mild cognitive impairment (MCI), General physicians (GPs), Interprofessional collaborations (IPC), KAP, India

## Abstract

**Background:**

Early identification and intervention of Mild Cognitive Impairment (MCI), led by General Physicians (GPs), can delay dementia onset and enhance patient outcomes. GPs recognize MCI risk factors, conduct assessments, and manage associated conditions, playing a crucial role in cognitive pathology intervention, especially in the era of Interprofessional Collaboration (IPC). In India, where cognitive impairment rates are projected to rise sharply, understanding GPs’ knowledge, attitudes, and practices (KAP) is vital. This study aimed to investigate the KAP of GPs regarding the diagnosis and treatment of MCI using a customized, predesigned questionnaire.

**Method:**

The study employed a cross-sectional design using a convenience sample of 180 invited participants between July and October 2023. A customized questionnaire, based on Lu et al., 2022, evaluated GPs’ KAP regarding MCI and IPC in the Indian context towards its assessment and management. The tool including 9 knowledge items, 15 attitude items, and 12 practice items, was made available through Google Forms and disseminated via WhatsApp. Responses were scored to indicate KAP levels, with maximum scores being 50 for knowledge, 75 for attitudes, and 60 for practices.

**Results:**

103 GPs completed the survey, showing varied practice experience. The average knowledge score was 28.1 ± 7.98, indicating uncertainty about MCI-related factors and diagnostic criteria. Attitude scores averaged 53.5 ± 4.73, with most GPs endorsing early detection and non-pharmacological interventions. Practice scores averaged 41.8 ± 8.32, showing mixed adherence to screening and referral practices. Most participants found IPC highly effective for MCI diagnosis and management, with many referring patients to specialists for confirmation, while over half used an IPC approach for both. Education level and previous experience significantly influenced knowledge and practice scores.

**Conclusion:**

This study sheds light on the evolving landscape of Indian GPs’ KAP related to MCI assessment and management. It identifies areas where understanding could be strengthened and highlight opportunities for growth through education and training. Notably, there is a need for increased involvement in IPC. These findings emphasize the importance of holistic approaches, advocating for enhanced education and the fostering of collaborative relationships across disciplines to tackle the rising prevalence of MCI in India effectively.

**Supplementary Information:**

The online version contains supplementary material available at 10.1186/s12875-025-02748-7.

## Background

The ‘GBD 2019 Dementia Forecasting Collaborators’ estimates that approximately 152.8 million individuals will have age-linked cognitive disorders, such as dementia, by 2050. Mild Cognitive Impairment (MCI) or mild neurocognitive disorder is a transitional stage between typical age-related cognitive changes and dementia [1–3]. It is a neurological condition found in aging individuals, that is characterized by subtle yet noticeable cognitive decline with minimal disruption of daily activities. Individuals diagnosed with MCI may either transition to major neurocognitive disorders such as dementia [4] or stabilize or revert to normal cognition at early stages [5, 6]. Considering the possibility of reversing MCI, a diagnosis of MCI should be viewed as an opportunity for early intervention rather than a definitive indicator of dementia [6]. Estimates indicate that delaying the onset of dementia by five years could reduce its prevalence by half [7]. However, research suggests that cognitive pathologies, spanning from MCI to severe dementia, are often underdiagnosed or overlooked in primary care settings [8, 9].

Early identification and intervention of MCI and dementia offers a range of benefits not only to patients and their families but also to communities at large at the national and global levels [10]. These benefits include, but are not limited to, differential diagnosis and ruling out other disorders, delayed or slowed progression of the disease, prompt access to medication, providing time for legal and financial planning, improved quality of life for individuals with cognitive impairment and their caregivers [11] and delayed institutionalization, thereby lowering the societal cost of healthcare [10]. General Physicians (GPs) are often the initial contacts for aging individuals with risk factors that could lead to MCI. They play a central role in the healthcare setting that allows them to gather further data on cognitive deterioration relative to a person’s prior level of cognitive function and identify a decline in cognitive capacity. GPs are usually the first to identify vascular disorders such as hypertension and diabetes [12–14], as well as other sensory aspect-related risk factors for MCI, such as hearing loss [15]. Additionally, they are also usually the first to be informed about any psychological symptoms and can therefore initiate and perform comprehensive biopsychosocial cognitive assessments [16]. GPs are responsible for facilitating appropriate referrals to specialists for confirmation of diagnosis and addressing the symptoms of MCI. Furthermore, GPs are well positioned for early intervention of the underlying vascular impairments that could lead to MCI. Managing these vascular conditions not only lowers the risk of MCI but also facilitates monitoring and controlling its progression. Moreover, early intervention by GPs can aid family members in anticipating and accepting their transition as future caregivers [17], thereby decreasing their caregiver burden [18].

The role of healthcare professionals, including GPs, in early identification and intervention for cognitive pathologies has become even more significant in the emerging era of Interprofessional Collaboration (IPC). IPC refers to the collaborative teamwork of healthcare professionals from various backgrounds who work collectively with patients, families, and communities to deliver optimal care across a range of healthcare environments [19]. IPC has been instrumental in the timely detection and management of disorders, leading to positive healthcare outcomes for individuals with different health issues [20–22]. Collaboration between GPs and other healthcare professionals, such as speech language pathologists (SLPs), pharmacists, and social workers, has shown high success rates in making an impact on the early identification and intervention of MCI [23, 24]. Following the diagnosis of MCI, the GP assumes the central role of a primary care professional to ensure effective and coordinated management of the disorder as well as to ensure that patient and caregiver needs are met [6]. Such collaborations that capitalize on unique skills among team members are not only appreciated and practiced but also advocated worldwide.

Recognizing the significant role that GPs play in the early identification of MCI and acknowledging the growing importance of IPC, it becomes essential to understand the current knowledge, attitudes, and behaviours of GPs about MCI. It cannot be denied that inadequate knowledge and attitudes, when put into practice, can unquestionably harm patients [7]. According to the theory of knowledge, attitude and practice (KAP), practice is influenced by both knowledge and attitude [25]. Individuals with higher levels of knowledge of MCI are more likely to have better attitudes towards the identification and management of MCI. The diagnostic decisions of GPs regarding dementia have been found to be impacted by their own perspectives on the condition and effectiveness of available treatments [26]. However, a lack of adequate knowledge among GPs regarding cognitive impairment has been shown to be a limiting factor for its detection and management [7, 27]. In a study conducted by Werner et al., a significant portion of the GPs had only heard of the term MCI while knowing almost nothing about it [28]. Similarly, Serrano et al. evaluated the opinions of GPs concerning MCI and found that the majority of the participants considered MCI to be an “ambiguous entity” and not a “predementia” stage [29]. Along with having poor knowledge, several GPs have also reported having little confidence in dementia care and management [27]. A meta-analysis on the ability of physicians to accurately detect MCI revealed that GPs face significant challenges in identifying individuals with MCI [30]. Most of the research has highlighted the significance of GPs’ KAP as a key determinant for fostering positive change in the future. While these studies were conducted locally, they suggest extending similar global initiatives to assess and enhance GPs’ KAPs at both the local and national levels, with a focus on enhancing culturally specific KAPs tailored to diverse demographics.

In the Indian context, a GP, also known as the “family doctor,” is usually the initial point of contact during times of illness. They are skilled at addressing a wide range of conditions and have expertise in various areas, such as physical examinations, emergency care, and primary healthcare [31]. The scenario of cognitive impairment in India is particularly concerning given the global perspective. Approximately 15–33% of people in India currently have MCI [32]. This number is expected to increase by 197% by 2050 [33]. In the background of worldwide initiatives to promote KAP regarding MCI among GPs, it is particularly crucial to assess the level of KAP among GPs in India. This becomes especially important considering the alarming projections for India. Addressing this issue may be even more significant in mitigating the impact of cognitive disorders in a timely manner. Furthermore, given the diversity of professionals involved in managing MCI in India, including neurologists, SLPs, social workers, and psychologists, there is great potential to explore the perspectives of primary care providers to effectively coordinate an interprofessional team since GPs are the central professionals involved. Such an investigation could highlight the existing conditions of diagnosis, treatment, and management strategies used for MCI patients in India. Hence, conducting a survey among these professionals can be pivotal in examining their understanding, practices, and attitudes towards MCI in India. Thus, the present study aimed to investigate the KAP of GPs regarding the diagnosis and treatment of MCI using a customized, predesigned questionnaire. The objectives of the study involved adapting and validating an existing questionnaire to suit the Indian context, followed by surveying the GPs using the adapted questionnaire.

## Methodology

The current study adopted a cross-sectional design and obtained approval from the Institutional Ethics Committee (IEC KMC MLR 05/2023/229). As an E-survey, the study adhered to the Checklist for Reporting Results of internet E-Surveys (CHERRIES) statement [34]. The study used a convenience sampling method, distributing an e-survey to practicing GPs who were members of specific WhatsApp groups accessible to the research team from July 2023 to October 2023. In the present study, GPs are operationally defined as practicing allopathic physicians with a minimum qualification of an MBBS degree and/or an MD in internal medicine by a medical college recognised by the National Medical Council of India. According to these criteria, the WhatsApp groups accessed by the research team contained a total of 180 practicing GPs.

### Adaption and validation of the KAP tool

The questionnaire utilized in the current study was adapted from Lu et al. (2022) [7]. Lu et al. (2022) had developed this questionnaire through an extensive literature review, focus group interviews, and Delphi consultations with experts which was validated based on content, indicator specification, and external validity [7]. Their questionnaire comprised of 55 items divided into four sections: sociodemographic details and work experience, knowledge, attitudes, and practices. The Knowledge section included eight questions covering MCI-related topics. Each question provided an “unsure” option to discourage guessing. Each correct answer scored as 1 and incorrect/unsure answers as 0. The Attitudes section contained 13 items assessing GPs’ perceptions of the condition, potential patient and societal responses, and necessary actions from healthcare providers. Responses on their questionnaire were rated on a five-point Likert scale ranging from “strongly disagree” (1 point) to “strongly agree” (5 points). The Practices section included questions measuring three domains of MCI: alerting (2 questions), confirming (4 questions), and managing (5 questions) of MCI.

Given that the original questionnaire was developed in a Chinese context, it was adapted and validated for the Indian context in the present study. The research team collectively reviewed each item in the questionnaire, customizing the language to suit Indian English and align it with the education and practices relevant to Indian GPs. Additionally, since the present study sought to understand GPs’ KAP in the context of IPC for assessing and managing MCI, the team incorporated relevant questions to assess these aspects. The specific modifications and additions made are detailed in the supplementary file 1. Once the team reached a consensus on linguistic accuracy and content appropriateness, the questionnaire was circulated to three experts, all GPs with over a decade of combined clinical and research experience. The experts assessed the items of the adapted questionnaire on a Likert scale ranging from 0 (very inappropriate) to 4 (very appropriate). The average scores given by the experts for each question were calculated, achieving a content validity index of 1, indicating excellent content validity for the developed questionnaire.

The final questionnaire, after development and validation, consisted of three sections: Knowledge, Attitudes, and Practices. The Knowledge section contained nine items about the prevalence, risk factors, screening, diagnosis, management, referral, prognosis, and conversion rate of MCI to dementia. Each correct choice was awarded 1 point, while incorrect and ‘unsure’ responses received 0 points, for a total possible score of 50. The Attitudes section included 15 items assessing opinions and beliefs about the identification, disclosure, and management of MCI, considering its impact on patients, families, doctors, and society. Responses were recorded on a Likert scale from “strongly disagree” (1 point) to “strongly agree” (5 points), with a maximum score of 75. The Practice section included 12 items across three domains: “alerting,” “confirming,” and “managing.” Responses were recorded on a Likert scale from “Never” (1 point) to “Always” (5 points), while the “Not Applicable” option, when selected, was scored as 1 point. The total possible score for this section was 60. Additionally, there were eleven questions related to the social demographics and work experience of the GPs that were not scored. The final questionnaire, as distributed to the participants, has been shared in the supplementary file 2. Scoring for each item and subscale is also provided in Supplementary File 2, along with the final questionnaire.

The questionnaire, in its final form, was converted into a Google Form, and trial runs were conducted among the investigators to check its feasibility and technical functionality before it was rolled out to the participants. The presentation of items was fixed on the Google form. The survey consisted of six pages, with each section starting on a new page. The number of questions per page ranged from 6 to 15. All questions were marked as mandatory, ensuring that participants were alerted if they missed any questions and preventing the survey from being completed without all answers provided. Participants had the option to return to previous pages to review their answers before finally submitting the questionnaire.

### Procedure

The final questionnaire, embedded in Google Forms, was circulated online as a voluntary survey with a common link through WhatsApp to certain GPs’ professional groups. The same link was circulated in each group three times, with 15-day intervals as reminders to participants willing to participate. Consent was obtained from the heads of the professional groups before the survey was circulated. Participants received a detailed explanation of the study, including its objectives and procedures. This information was included in the Google form for participants to review before they chose to participate in the survey. Informed consent was obtained before the participants’ participation in the study. Participants could only respond once using the Google form. To ensure confidentiality, no names or email addresses were recorded. All questions were mandatory, and responses could not be modified after submission. Each section of the survey included directions and definitions of relevant terms to guide the participants. Participants were informed that the average time to complete the questionnaire would be 8–10 min. They were also notified that their responses would be automatically stored on Google Drive and could only be accessed by the authors. The participants were thanked for their participation and were offered no incentives.

### Data analysis

The obtained data was analysed statistically for frequency distribution. The mean and standard deviation for each of the three sections were also computed and compared among participants with different characteristics using either Student’s t-test or ANOVA. To explore significant differences further, a post hoc pairwise analysis was conducted. The statistical analysis was conducted utilizing Jamovi software.

## Results

A total of 180 GPs from various healthcare settings in India received the survey link. Of these, 150 viewed the survey, and 103 responded to it. Among the respondents, 77.7% were male, and approximately 44.0% were under the age of 30 years. All respondents practiced allopathy, with the majority of participating GPs holding either a Doctor of Medicine (MD) or a Diploma of National Board (DNB) qualification in internal medicine. Furthermore, 2% held additional degrees such as fellowships in fundamental critical care support and super specialization in nephrology. Majority of the GPs worked in a medical college setting, with nearly half having less than five years of work experience. Most GPs saw up to 50 patients per day, while approximately 3.0% attended to more than 100 patients daily. Although few patients sought help for memory complaints, a significant number (77.7%) of patients reported having cardiovascular risk factors (CVRFs). Additionally, most respondents reported being uncertain or lacking experience and training regarding MCI. The results are depicted in Tables 1 and 2.

**Table 1:** Sociodemographic characteristics of general physicians

Sociodemographic Variables	Percentage (%)
**Gender**	
Male	77.7
Females	22.3
**Age (years)**	
< 30	43.7
30–39	35.0
40–49	13.5
> 50	7.8
**Degree**	
MBBS	10.6
MD/DNB (Internal Medicine)	87.4
Others (clinical fellowships and super specializations)	2.0
**Location (Clinical Practice)**	
North India	4.8
South India	84.6
East India	1.9
West India	6.8
Central India	1.9

The table summarizes the sociodemographic characteristics of the respondents, including gender distribution, age groups, educational degrees and location of clinical practice

**Table 2:** Work experience characteristics of general physicians

Work experience variables	Percentage (%)
**Work set-up**	
Medical college	58.3
Private practice	13.6
Government service	10.7
Corporate hospital	17.4
**Work experience (years)**	
< 5	50.5
5–9	18.4
10–14	12.7
*≥* 15	18.4
**Average number of patients treated on a daily basis**	
< 20	26.2
20–50	61.2
50–99	9.7
> 100	2.9
**Proportion of patients with memory issues**	
0	5.8
< 10%	71.8
10–29%	14.6
*≥* 30	3.9
Unsure	3.9
**Proportion of Patients with CVRFs**	
0	1.0
< 10%	4.8
10–29%	16.5
*≥* 30	77.7
**Experience with MCI**	
Yes	38.8
No	42.7
Unsure	18.5
**Training to detect and manage MCI**	
Yes	43.7
No	40.8
Unsure	15.5

The table provides percentages of general physicians based on their work set-up, years of work experience, average number of patients treated daily, proportion of patients with memory issues and cardiovascular risk factors (CVRFs), experience with Mild Cognitive Impairment (MCI), and training to detect and manage MCI

The average knowledge score was 28.1 (SD = 7.98) out of a possible 50. The majority of respondents were either incorrect (64.1%) or unsure (20.4%) about the prevalence of MCI in individuals with CVRFs. While most participants correctly identified various risk factors associated with MCI, only 32.0% recognized cognitive engagement as a factor linked to MCI occurrence. Additionally, the majority of GPs did not consider hearing loss (43.7%), education level (40.8%), or employment status (38.8%) to be contributing risk factors for MCI. Knowledge of the prevalence of MCI and progression to dementia among GPs were generally inadequate, with many GPs providing incorrect answers (34.0%) or expressing uncertainty (26.2%). Although 66.6% of participants correctly identified the involvement of various healthcare professionals in the diagnosis of MCI, only 27.2% recognized the role of speech-language pathologists (SLPs). The majority of participants were also unaware or uncertain about the various diagnostic criteria (97.1%), screening tools (96.1%), and diagnostic tools (96.1%) available for evaluating MCI. Most respondents acknowledged cognitive training (84.5%), social activities (81.6%), yoga (81.6%), musical intervention (68.0%), and aerobic exercise (63.1%) as nonpharmacological intervention methods for managing MCI, while only 45.6% recognized the role of the Mediterranean diet. The results are depicted in Table 3.

**Table 3:** Frequency of responses for items related to knowledge of general physicians about mild cognitive impairment

	Correct (%)	Incorrect (%)	Unsure (%)
**1. Prevalence of MCI in individuals with CVRF**	15.5	64.1	20.4
**2. Risk factors associated with cognitive decline (Total)**	7.8	91.2	1.0
Hypertension	75.7	24.3	-
Diabetes Mellitus	88.3	11.7	-
Hyperlipidaemia	68.9	31.1	-
Family History	72.8	27.2	-
Lack of exercise	59.2	40.8	-
Alcohol or drug abuse	87.4	12.6	-
Smoking	72.8	27.2	-
Stressful lifestyle	81.6	18.4	-
Cognitive engagement	32.0	68.0	-
Sleeping habits	73.8	26.2	-
Hearing loss	43.7	56.3	-
Depression	79.6	20.4	-
Education level	40.8	59.2	-
Employment status	38.8	61.2	-
**3. HCPs involved in MCI diagnosis (Total)**	66.1	33.9	-
Physician	50.5	49.5	-
SLP	27.2	72.8	-
Psychologist	33.0	67.0	-
Psychiatrist	42.7	57.3	-
Neurologist	47.6	52.4	-
All of the above	66.0	34.0	-
**4. MCI conversion rate to Dementia**	39.8	34.0	26.2
**5. MCI diagnostic criteria (Total)**	2.9	60.2	36.9
Petersons criteria	17.5	82.5	-
DSM-V criteria	49.5	50.5	-
NIAAA criteria	25.2	74.8	-
MCI: Manchester approach	21.4	78.6	-
**6. Commonly used MCI screening scales (Total)**	3.9	86.4	9.7
MoCA	44.7	55.3	-
MMSE	81.6	18.4	-
ACE-R	13.6	86.4	-
ICMR-NCTB	16.5	83.5	-
CDR	13.6	86.4	-
ADL	23.3	76.7	-
GPCOG	19.4	80.6	-
**7. Treatment of MCI**	40.8	43.7	15.5
**8. Diagnostics tool for MCI**	6.8	91.3	1.9
Neuropsychological assessment	72.8	27.2	-
Neuropsychiatric assessment	73.8	26.2	-
Thyroid-stimulating hormone (TSH), triiodothyronine (T3), and free thyroxine (FT4)	83.5	16.5	-
Vitamin B12 serum	80.6	19.4	-
Serum Folic acid	43.7	56.3	-
Venereal Disease Research Laboratory Test (VDRL)	49.5	50.5	-
Computed tomography (CT) of the brain	19.4	80.6	-
Magnetic resonance imaging (MRI) of the brain	56.3	43.7	-
Single photon emission computed tomography (SPECT) scan of the brain	14.6	85.4	-
HIV	59.2	40.8	-
**9. Non-pharmacological interventions for MCI**	31.1	62.1	6.8
Aerobic exercise	63.1	36.9	-
Mediterranean diet	45.6	54.4	-
Music	68.0	32.0	-
Social activities	81.6	18.4	-
Yoga	81.6	18.4	-
Cognitive training	84.5	15.5	-

The table presents the frequency of correct, incorrect, and unsure responses among respondents regarding their knowledge of MCI (Mild Cognitive Impairment) and its associated risk factors, diagnosis, screening scales, treatment, diagnostic tools, and non-pharmacological interventions

**Table 4:** Frequency of responses for items related to attitude of general physicians towards mild cognitive impairment

Attitude Items	Strongly Agree (%)	Agree (%)	Unsure (%)	Disagree (%)	Strongly Disagree (%)
Mild Cognitive Impairment is not a disease, but a typical aging process	4.9	35.9	11.7	43.7	3.9
There are more advantages than disadvantages to finding out if someone has Mild Cognitive Impairment.	38.8	48.5	7.8	2.9	1.9
All patients suspected of Mild Cognitive Impairment should undergo a diagnostic evaluation.	47.6	45.6	5.8	1.0	-
Early recognition and management of Mild Cognitive Impairment can delay the progression to Dementia	41.7	50.5	7.8	-	-
There are more advantages than disadvantages in managing individuals with Mild Cognitive Impairment using pharmacological methods	10.7	31.1	37.9	19.4	1.0
There are more advantages than disadvantages in managing individuals with Mild Cognitive Impairment using nonpharmacological methods	34.0	45.6	15.5	3.9	1.0
Patients with dementia can be a drain on medical and social resources.	27.2	40.8	12.6	17.5	1.9
Disclosure of Mild Cognitive Impairment could cause stress and frustration to the patients.	15.5	66.0	7.8	9.7	1.0
Disclosure of Mild Cognitive Impairment could cause stress and frustration to the families.	13.6	68.0	8.7	9.7	-
Disclosure of Mild Cognitive Impairment to the patients could cause embarrassment or discomfort to doctors.	5.8	21.4	9.7	48.5	14.6
Being diagnosed with Mild Cognitive Impairment could provide some hope for patients compared to being diagnosed with Dementia.	29.1	57.3	9.7	3.9	-
Mild Cognitive Impairment detection and management provides no economic benefits to the society.	4.9	9.7	17.5	48.5	19.4
It is responsibility of the doctor to recognize Mild Cognitive Impairment in the primary care setting.	33.0	53.4	7.8	4.9	1.0
It is responsibility of the doctor to manage Mild Cognitive Impairment in the primary care setting.	23.3	43.7	17.5	15.5	-
In the diagnosis and management of Mild Cognitive Impairment, an Interprofessional Collaborative (IPC) approach would be highly effective	52.4	41.7	4.9	-	1.0

The table presents the frequency of attitudes among respondents regarding various aspects of Mild Cognitive Impairment (MCI), including its nature, the benefits and drawbacks of diagnosis and management, the role of healthcare providers, and the effectiveness of different approaches

The average attitude scores out of a total of 75 was 53.5 (SD = 4.73). Approximately 40.8% of respondents perceived MCI as a natural consequence of aging, while fewer than half of the participants (47.6%) considered MCI to be a disorder. An overwhelming majority (92.2%) believed that early detection of MCI might postpone the onset of dementia, and a similar percentage (93.1%) expressed readiness to recommend diagnostic evaluations for suspected cases of MCI. Additionally, a significant proportion (79.6%) favoured nonpharmacological interventions over pharmacological interventions for MCI management. Concerning the impact of diagnosis, a substantial portion (86.4%) agreed that an MCI diagnosis could offer hope compared to a diagnosis of dementia. However, 81.6% expressed concerns about potential stress and frustration resulting from MCI disclosure for patients and their families. While 68.0% considered dementia patients to be potential burdens on resources, nearly the same percentage (68.0%) expressed disagreement with the assertion that there were no economic benefits to society from detecting and managing MCI. In the diagnosis and management of MCI, the majority of participants believed it was the responsibility of doctors to identify (86.4%) and manage (67.0%) MCI. Additionally, 94.1% of participants believed that an IPC approach would be highly effective. The results are depicted in Table 4.

**Table 5:** Frequency of responses for items related to practice of general physicians with mild cognitive impairment

Practice Items	Always (%)	Usually (%)	Sometimes (%)	Seldom (%)	Never (%)	Not Applicable (%)
I take symptoms of memory loss as the criteria for Mild Cognitive Impairment detection.	20.4	51.5	20.4	4.9	2.9	-
I take psychiatric symptoms as the criteria for Mild Cognitive Impairment detection.	8.7	30.1	31.1	20.4	8.7	1.0
I generally ask if a patient has family history of Dementia	46.6	26.2	17.5	5.8	3.9	-
I usually screen for Mild Cognitive Impairment in individuals with cardiovascular risk factors (Diabetes, Hypertension, Dyslipidemia, Obesity, Smoking)	21.4	28.2	24.3	16.5	9.7	-
I usually utilize a screening scale for detection of Mild Cognitive Impairment.	14.6	28.2	24.3	18.4	13.6	1.0
I usually refer a suspected individual with Mild Cognitive Impairment to a specialist for final diagnosis.	34.0	33.0	23.3	7.8	1.9	-
I usually discuss the probable diagnosis of Mild Cognitive Impairment with the patient	12.6	35.9	30.1	10.7	10.7	-
I usually discuss the probable diagnosis of Mild Cognitive Impairment with the patient’s family.	34.0	35.0	19.4	8.7	2.9	-
I take an Interprofessional Collaborative (IPC) approach to diagnose Mild Cognitive Impairment.	27.2	31.1	24.3	8.7	4.9	3.9
I take an Interprofessional Collaborative (IPC) approach to manage Mild Cognitive Impairment.	29.1	35.0	20.4	10.7	2.9	1.9
I usually provide nonpharmacological interventions for treatment of Mild Cognitive Impairment.	15.5	39.8	28.2	5.8	6.8	3.9
I usually prescribe medications for treatment of Mild Cognitive Impairment.	4.9	15.5	34.0	26.2	15.5	3.9

The table presents the frequency of various practices among respondents in detecting, diagnosing, and managing Mild Cognitive Impairment (MCI), including the use of criteria, screening scales, interprofessional approaches, and treatment methods

**Table 6:** Factors associated with Knowledge attitudes and practices regarding mild cognitive impairment among General Physicians

Characteristics	Knowledge			Attitude			Practice		
	Mean	SD	ANOVA/t-test	Mean	SD	ANOVA/t-test	Mean	SD	ANOVA/t-test
**Age (Years)**			F (3,24) = 0.05, *p* = 0.98			F (3,25) = 1.24, *p* = 0.31			F (3,27.7) = 1.31, *p* = 0.29
< 30	28.2	7.1		52.6	4.9		41.2	8.8	
30–39	27.8	8.7		53.7	4.3		41.2	8.6	
40–49	28.6	8.7		55.6	5.2		43.5	6.9	
*≥* 50	27.4	9.6		54.3	4.5		45.6	6.2	
**Gender**			t (101) = 0.27, *p* = 0.78			t (101) = -0.12, *p* = 0.90			t (101) = 1.72, *p* = 0.08
Male	28.2	8.2		53.5	4.9		42.6	8.5	
Female	27.7	7.1		53.7	3.9		39.2	7.3	
**Education**			**F (2, 14.7) = 24.97**, ***p*** < 0.001*			F (2,2.5) = 1.52, *p* = 0.36			F (2,2.5) = 1.47, *p* = 0.37
MBBS	25.7	8.4	*MBBS vs. MD/DNB: *p* = 0.57 *MBBS vs. Others: *p* = 0.27 *MD/DNB vs. Others:*p* < **0.001**	51.7	3.7		37.0	9.3	
MD/DNB	28.5	7.9		53.9	4.7		42.3	8.1	
Others	21.5	0.7		49.0	7.1		45.0	7.1	
**Work Setup**			F (3,29.4) = 1.54, *p* = 0.22			F (3,28) = 0.30, *p* = 0.81			F (3,28.4) = 0.66, *p* = 0.58
Medical College	29.5	8.1		53.5	4.9		41.5	8.6	
Private Practice	26.6	5.9		52.9	4.6		42.0	8.3	
Government Service	25.8	6.8		54.7	4.9		44.9	7.7	
Corporate Hospital	25.9	9.0		53.6	4.5		40.9	7.9	
**Experience (Years)**			F (3,33) = 0.31, *p* = 0.81			F (3,35.2) = 1.70, *p* = 0.18			F (3,36.6) = 0.73, *p* = 0.53
< 5	28.1	7.3		52.8	4.8		42.0	8.7	
5–9	26.7	7.5		53.1	4.3		40.0	8.9	
10–14	29.9	10.5		56.0	5.4		41.0	7.3	
*≥* 15	28.1	8.8		54.5	4.1		43.7	7.2	
**Daily Visits**			F (3,8.2) = 0.67, *p* = 0.59			F (3,8.5) = 0.25, *p* = 0.85			F (3,11.2) = 1.37, *p* = 0.30
< 20	29.1	8.3		53.9	4.6		41.9	8.8	
20–50	28.1	7.6		53.5	4.8		41.7	8.7	
50–99	24.5	8.3		53.5	4.9		43.4	5.2	
> 100	30.0	12.1		51.3	4.6		38.3	3.1	
**Proportion of patients with memory related issues seen in last month**			F (4,9.56) = 1.075, *p* = 0.42			F (4,9.8) = 1.277, *p* = 0.34			F (4,9.1) = 0.24, *p* = 0.90
0	28.5	7.01		56.2	5.1		41.0	10.2	
*≤* 10%	27.5	8.3		53.6	4.7		41.6	7.8	
10–29%	31.3	5.9		53.0	5.2		43.3	9.9	
*≥* 30%	29.3	6.7		54.8	3.5		44.5	7.8	
Unsure	24.0	10.7		50.3	3.3		38.5	12.1	
**Proportion of patients with CVRF seen in last month**									
0	30.0	NaN		57.0	NaN		49.0	NaN	
*≤* 10%	22.2	7.6		51.8	3.4		42.0	12.8	
10–29%	25.6	7.7		52.1	3.5		44.5	8.8	
*≥* 30%	28.9	7.9		53.9	4.9		41.1	7.9	
**Experience with MCI**			F (2,49.3) = 1.89, *p* = 0.16			F (2,49.3) = 0.12, *p* = 0.88			**F (2,45.2) = 7.53**, ***p*** = 0.001*
Yes	29.9	8.0		53.3	4.5		45.1	6.2	*Yes vs. No:*p* < **0.001** *No vs. Unsure: *p* = 0.42 * Yes vs. Unsure: *p* = 0.39
No	26.5	7.8		53.8	4.9		38.8	8.5	
Unsure	28.0	7.8		53.5	4.7		41.9	9.3	
**Training to detect and manage MCI**			F (2,41.3) = 2.41, *p* = 0.10			F (2,42.9) = 0.72, *p* = 0.49			**F (2, 40.4) = 8.43**, ***p*** < 0.001*
Yes	29.8	7.7		53.0	5.0		45.2	7.2	*Yes vs. No:*p* < **0.001** *No vs. Unsure: *p* = 0.59 *Unsure vs. Yes: *p* = 0.18
No	27.5	7.9		53.8	4.5		38.5	8.1	
Unsure	24.8	8.2		54.6	4.5		40.9	8.5	

*Post-hoc analysis

BOLD represents Significant difference

NaN: Not enough observations

The table presents the factors associated with Knowledge, Attitudes, and Practices (KAP) scores regarding Mild Cognitive Impairment (MCI) among General Physicians (GPs). The table shows mean scores, standard deviations (SD), and statistical values (F/t and P) for various demographic and professional characteristics. Post hoc analysis results are also provided where significant differences were found, indicating pairwise comparisons among groups to identify specific differences

The average practice score was 41.8 (SD = 8.32) out of a possible 60. More than half of the participants (71.9%) were alerted to and identified memory problems as one of the first signs of MCI, while only 38.8% consistently recognized psychiatric symptoms as indicators of MCI. Additionally, the majority (72.4%) reported regularly inquiring about family history regarding dementia. Approximately half (49.6%) of the respondents screened for MCI were individuals with CVRFs, and only 42.0% used scales for screening purposes. Approximately 67.0% of the participants referred patients to specialists for confirming an MCI diagnosis; however, over half of the participants employed an IPC approach to both diagnose (58.0%) and manage (64.0%) MCI. Regarding the disclosure of a probable MCI diagnosis, a substantial proportion (48.5%) of GPs always or usually, or sometimes (30.1%), discussed the diagnosis with the patient, while they always or usually (69.0%) or sometimes (19.4%) discussed the diagnosis with the patient’s family. Regarding managing MCI, 55.3% of participants provided nonpharmacological interventions, while 20.4% of the participants prescribed medications. The results are depicted in Table 5.

### Factors associated with knowledge, attitudes, and practices

One-way ANOVA and Student’s t-tests were conducted to compare the means of participants with different characteristics. Following the main analysis, a post hoc analysis using the Games-Howell test was performed to identify significant differences between groups. Education levels were found to be significantly associated with the knowledge scores [F(2, 14.7) = 24.97, *p* < 0.001]. The post hoc analysis revealed that participants with MD/DNB qualifications scored significantly higher than those in the ‘Others’ category (*p* < 0.001). Additionally, prior experience of the participants in diagnosing and managing MCI was found to be associated with the practice scores [F(2, 45.2) = 7.53, *p* = 0.001]. The post hoc analysis indicated that those with prior experience with MCI scored significantly higher on practice scores than those without prior experience (*p* < 0.001). A similar association was found between the training history of the participants and their practice scores [F(2, 40.4) = 8.43, *p* < 0.001], where those who had received any form of training to manage MCI obtained significantly higher practice scores than those who had not undergone any additional training for MCI (*p* < 0.001), as detailed in Table 6.

## Discussion

The present study explored the perspectives of general physicians (GPs) towards the assessment and management of mild cognitive impairment (MCI) within the Indian context. Understanding the viewpoints of community health professionals who are vital in addressing the growing population of individuals living with dementia is crucial. The findings of this study indicate that GPs in India may have some gaps in their knowledge and attitudes, as well as potential areas for improvement in their clinical practices in regard to identifying and managing MCI within the community.

GPs worldwide have been reported to possess a concerning deficit in their knowledge and understanding of MCI. The average knowledge level of the participants in the current study was generally indicated by low scores among GPs. Over 80.0% of the participants were either incorrect or uncertain regarding the prevalence rate of MCI in individuals with cardiovascular risk factors (CVRFs). Similarly, a German study found that GPs tended to underestimate the presence of MCI in their patients [35]. A Hungarian study also revealed that GPs had low awareness and knowledge about the epidemiology of MCI, as nearly half (44.9%) of the respondents were unaware of MCI [36]. Interestingly, a study by Werner et al. showed that while almost one-third of their participants were unfamiliar with or had only heard about MCI as a medical terminology, more than half (59.1%) correctly identified the contribution of cardiovascular diseases to Alzheimer’s disease. In this regard, it becomes imperative to recognize the significance of GPs in the healthcare system as primary care providers. Understanding the manner in which knowledge is conveyed to GPs is crucial for comprehending the root cause of their insufficient knowledge.

The observed knowledge gap could be attributed to the prevalent educational paradigm in India. In contrast to medical schools in the UK, which use clinical rotations, lectures, seminars, case studies and home/community visits for training in dementia care [37], the Indian undergraduate medical curriculum may not cover this comprehensively. Until 2019, when the majority of the GPs in the present study completed their undergraduate education, dementia was typically addressed under the broader umbrella of “Common problems in geriatrics,” in General Medicine where it was covered alongside other conditions rather than as a separate subject. Only three sessions were dedicated to the topic of Clinical Geriatrics with MCI often receiving only brief mention of this topic. In the psychiatry curriculum, dementia was addressed under the topic of “Organic mental disorders.” Postgraduate training in internal medicine provides exposure to dementia care under the general purview of clinical rotations in medical wards and during neurology rotations. With teaching largely focused on ‘localization’ in neurological disorders, care, prevention and rehabilitation of dementia often finds scant mention even within the neurology rotation. There could be ample opportunity to enhance medical education and better prepare healthcare professionals to address conditions such as MCI and dementia in the future. Similar sentiments were reported in a recent study that emphasized that the current medical education program in India may not fully cover cognitive impairments such as MCI and dementia but may provide an opportunity to improve training for future healthcare professionals [38]. It was seen that despite training in dementia during the study [38], while students demonstrated a fundamental understanding of dementia and related conditions after the sensitization program, they still continued to have a limited understanding of the associated risk factors, prevention and care for dementia patients. It is imperative that medical undergraduate academic curricula emphasize the significance of dementia care and management [38]. Notably, the current study showed that practitioners with higher levels of education (postgraduation and above) possess better knowledge about the assessment and management of MCI, aligning with the idea that education could be one of the important prerequisites for optimizing knowledge. The post hoc analysis revealed a significant difference between the MD/DNB group and the ‘Others’ group. However, this finding cannot be generalized, since the ‘Others’ group made up just 2.0% of the study participants. The respondents of the current research have studied the previous curriculum. Since then, Curriculum-Based Medical Education (CBME) has been introduced in India in 2019 and has been updated to increase the focus on dementia-specific education, although not particularly focusing on MCI. This advancement is likely to positively influence the knowledge of emerging physicians in the country, equipping them with enhanced knowledge regarding dementia and related disorders.

The participants in the current study also demonstrated a limited understanding of both the modifiable and nonmodifiable risk factors associated with MCI. While most participants acknowledged the influence of family history of dementia and CVRFs on MCI, they seemed to overlook significant risk factors such as hearing loss, educational level, and cognitive engagement. Vascular cognitive impairment (VCI) is a milder cognitive impairment stemming from cerebrovascular disease. Recent findings indicate that modifying vascular risk factors and lifestyle choices can lower dementia risk by as much as 40.0%, with hearing loss accounting for 8.0% and low education accounting for 7.0% [6, 39]. Consistent with these findings, participants in the present study showed limited awareness regarding the progression from MCI to dementia or its reversal back to normal cognition. Approximately half of the individuals diagnosed with MCI either maintain stability or improve their condition toward normal cognition, provided they are able to keep a check on the CVRFs [40]. Several studies have confirmed these results. Rochette et al. reported a 48.9% reduction in MCI frequency following bariatric surgery in individuals with severe obesity [41]. Furthermore, Katayama et al. observed a 33.3% reversal rate from MCI to normal cognition through multidomain lifestyle activities, while Shimada et al. demonstrated greater odds of MCI reversion among those participating in driving, gardening, sports, hobbies, and community meetings [42, 43]. In the Indian context, despite extensive national public health initiatives focusing on identifying and addressing noncommunicable diseases such as diabetes, hypertension, and stroke within local communities, there remains an opportunity for proactive exploration into how effectively managing these conditions could positively influence the risk of developing cognitive pathologies, spanning from MCI to severe dementia. In this regard, it is also worthy to note that pathways for dementia care in India is heavily dependent on expensive and highly specialised tertiary level private healthcare. Most of this private care has no link with the primary, secondary and tertiary level care provided by government agencies. The service gap for dementia care in India is about 90.0%, with less than one in ten dementia patients receiving any degree of diagnosis, treatment or care [44]. The majority of the GPs surveyed in this study were from the private medical sector and their attitudes and lack of awareness of cognitive impairment is largely reflective of the prevalent norms regarding dementia care in India.

The level of knowledge GPs possess regarding MCI significantly influences their attitude to positively engage in its identification and management. Approximately half of the participants surveyed in the present study were unsure whether MCI is classified as a disorder or is part of the natural aging process. These trends align with previous research where participants struggled to differentiate between MCI resulting from a disorder or as a consequence of normal aging [35, 45]. Werner et al. reported that 71.7% of physicians surveyed believed that MCI stems from typical aging processes [28]. However, a promising indication emerged, as more than 50.0% of the participants in the present study expressed their support for timely identification and management of MCI. While the majority of GPs acknowledged that disclosing a diagnosis of MCI could lead to stress and frustration for patients and their families, they generally did not report feeling embarrassed or uncomfortable about such disclosures. Many GPs believed that timely disclosure of MCI offers patients more hope than does a diagnosis of dementia. Early identification of MCI can potentially delay progression to dementia and aid in future risk reduction planning for both patients and their families. Additionally, it provides GPs with the opportunity to enroll such patients in clinical trials and plan regular follow-ups [46]. However, some studies have shown that GPs may often perceive giving a diagnosis of dementia as potentially harmful to family members who may not be ready to accept the reality [47–49]. We believe that providing specific training to our aspiring GPs, coupled with appropriate counselling skillsets, could effectively alleviate these concerns.

In the current study, a majority of GPs exhibited a clear preference for nonpharmacological interventions over pharmacological strategies for managing MCI. These findings are consistent with a Swiss study in which approximately 45.0% of participants disagreed with the effectiveness of anti-dementia drugs [50]. Numerous studies have demonstrated that GPs tend to support and recommend nonpharmacological treatments, such as cognitive training, dietary changes, and physical and social activities, more frequently than pharmacological options [28, 35, 51]. Hungarian GPs expressed confidence in prescribing cognition-enhancing drugs for mild-stage dementia, believing in their potential to slow disease progression despite acknowledging diminishing effectiveness as dementia severity increases [36]. Most of the GPs in the present study disagreed that detecting and managing MCI does not yield economic benefits for society. The results from the Vietnamese study as well as the Chinese study aligned with the attitudes of the GPs from the current study [7, 27]. Research indicates that identifying MCI early on not only offers economic advantages to the healthcare system but also yields numerous other benefits. Given the global burden of Alzheimer’s disease and other dementias, coupled with shortages in primary healthcare providers for an aging population, early detection and diagnosis of MCI can improve overall health and quality of life by enhancing the management of other medical conditions, fostering comprehensive care, and promoting better health outcomes. Furthermore, closely monitoring cognitive levels and administering appropriate treatments can help mitigate the progression to dementia [19].

Consistent with the scores observed in the knowledge and attitude sections, GPs demonstrated limited performance in the practice domain. Lower scores in the practice domain may be attributed to inadequate knowledge and generally inconsistent attitudes toward assessing and managing MCI. It was observed that the majority of the GPs were alerted by memory loss as a sign of MCI, while there were more scattered responses for those who were alerted by any psychiatric symptom. This result mirrors the findings of a cross-sectional investigation conducted in Germany and Sweden, where GPs were able to identify MCI in only a small number of patients based solely on clinical impressions [35, 52]. Approximately 40.0% of the participants in the Chinese study considered memory loss or psychiatric symptoms to be indicative of MCI [7], in contrast with 63.1% of the Hungarian GPs who identified memory complaints as symptoms of MCI. Such discrepancies could be addressed by implementing standardized diagnostic criteria for uniformly identifying MCI and providing training to GPs to enhance their ability to recognize and manage MCI effectively. To ensure that individuals with MCI receive prompt and effective intervention, a conclusive diagnosis is crucial. Regrettably, only 42.8% of the participants in this study usually or always used a screening scale to detect MCI, compared to 24.3% of GPs who sometimes employed these scales. This disparity may be attributed to limited awareness among GPs regarding the range of available screening tools. Evaluating the feasibility and effectiveness of various screening tools for improving diagnostic accuracy and facilitating early intervention could help in propagating guidelines for MCI detection in primary care settings. Although family history is routinely assessed during MCI evaluations by GPs, fewer than half of individuals with any CVRF are screened for MCI. Given the significance of CVRFs as major risk factors for MCI and considering India’s high prevalence of vascular dementia, there is an opportunity for GPs to act by using the diagnosis of any CVRFs as an opportunity to also screen for MCI. However, it is a promising indicator that more than half of the participants would seek a specialist’s opinion for a conclusive diagnosis in suspected cases of MCI. The results of a Chinese study reported similar findings in terms of MCI screening and referrals for final diagnosis [7]. In line with the results from the Chinese and German studies, the GPs in the present research were more comfortable sharing the probable diagnosis of MCI with the patient’s family rather than with the patient themselves [7, 35]. This reflects the general practice of collusion prevalent in oriental cultures such as India [53]. It is worth noting that in the present study, GPs with prior experience and training in recognizing and managing MCI demonstrated higher practice scores compared to those without such qualifications. Training is a widely adopted method globally for improving professional competence and upholding high standards. Similarly, previous research has indicated that offering training in dementia prevention and care can enhance the knowledge and practices of GPs, thereby equipping them as workforce for the future [27].

Another important aspect in the context of the present study is the shift in the landscape of research into the pharmacological treatment of dementia, from symptom relief to disease modification [54]. Newer drugs, such as lecanemab and donanemab, recently approved by the FDA, target amyloid modulation in the brain and may actively prevent the progression of MCI to advanced dementia. These and other disease-modifying drugs currently in late-stage clinical development may herald a paradigm shift in the approach to early detection of cognitive impairment [54, 55]. Disease-modifying therapies may not benefit patients in the advanced stages of dementia. However, these therapies may bring into focus various types of patients in the early stages of cognitive impairment or those at risk of cognitive decline, who could benefit from early identification [56]. The global healthcare workforce is currently reported to be inadequately equipped to handle this new and large cohort of patients, most of whom are undetected by the health system [57]. Although many of these patients may be referred to specialists in cognitive impairment, the sheer volume of such cases may necessitate that primary care physicians be better trained and more aware of methods to screen and identify patients with MCI who may benefit most from these new therapies [57].

## Promise of interprofessional collaboration for mild cognitive impairment

The latest research explored GPs’ attitudes and actions regarding interprofessional collaboration (IPC) in response to the increasing demand for care for individuals with cognitive disorders. IPC has emerged as a pivotal strategy for swiftly identifying and addressing conditions, leading to positive healthcare outcomes for patients across numerous health issues. IPC has been shown to be effective in enhancing MCI management and the quality of life of patients through collaboration between various specialists, including physicians, neurologists, SLPs, social workers, psychiatrists, psychologists, nurses and pharmacists [6, 23, 28], to improve MCI and dementia detection and multifaceted interventions, resulting in cognitive enhancements for patients [58, 59]. Although the majority of the respondents in the current study had knowledge about various healthcare professionals involved in the care and management of MCI, it was seen that only 27.2% knew about the involvement of SLPs. SLPs can offer counselling, collaboration, and prevention services, leveraging their communication expertise to support care teams and improve patient and family outcomes. The majority of GPs commonly view the diagnosis of MCI as falling under the purview of specialists [48]. Collaboration with specialists can provide valuable insights, optimize diagnostic processes and improve patient outcomes. Moreover, given the complexity of treatment options available for MCI, there is a need for a collaborative model for optimizing MCI care delivery and improving patient outcomes. Research in Vietnam highlighted that less than 50.0% of GPs endorsed nonpharmacological approaches for dementia management, emphasizing the need for specialist involvement in diagnosis. Against the backdrop of the perceptions of MCI being a drain on resources, employing an IPC approach has become increasingly imperative. IPC benefits patients and healthcare infrastructure by pooling expertise for early detection and patient-centric interventions, ensuring efficient resource use and improved health outcomes. It enhances the access, coordination, and safety of services, reducing treatment duration and costs while boosting satisfaction and acceptance. International studies from Vietnam, China and Scotland echo the importance of such collaboration in managing MCI [7, 27]. In the Indian context, where dementia cases are on the rise, there is a notable gap in emphasizing IPC, particularly concerning MCI care. Collaborative efforts among specialists hold immense potential. Introducing IPC teams could ease GPs’ workload by facilitating early diagnosis and management of MCI patients, allowing GPs to focus on the diagnosis of CVRFs, while the IPC team handles cognitive screening and promotes engagement. Prioritizing collaboration and holistic patient care also aligns with the broader goal of promoting good health and well-being, as outlined in the UN Sustainable Development Goal 3. By prioritizing collaboration and patient-centric care, primary care practices can significantly enhance health outcomes and quality of life for individuals affected by neurodegenerative conditions such as MCI and dementia.

## Limitations and future directions

The study was conducted using a convenience sampling approach within a limited cohort, mostly from southern parts of India, as the e-survey was distributed exclusively among specific WhatsApp groups accessible to the investigating team. This approach poses potential response bias and limits the generalizability of the findings to the broader Indian context. Future studies could incorporate more robust sampling methods with a larger and geographically well-represented cohort of GPs. Additionally, the present study did not capture the experiences GPs might have gained in cognitive disorders during their residency. This data could potentially skew the research findings. Future studies could explore the association of this aspect with the KAP of GPs regarding MCI. Furthermore, most respondents in the present research worked at medical colleges, which are tertiary-level care facilities. It would be valuable for future researchers to examine the KAPs of GPs from other healthcare pathways in India. It is possible that the respondents in this study may have confused IPC with multidisciplinary care, despite a disclaimer accompanying each IPC-related question and clarifying its meaning. Studies specifically examining KAP for interprofessional care compared to multidisciplinary care could clarify this in future research. This concern arises because despite good perception scores regarding IPC among GPs in the present study, their actual engagement in IPC remains relatively low at the ground level. Both practical implementation and research on IPC, generally and specifically for neurodegenerative conditions such as MCI, are limited in the Indian context. Therefore, it is essential to delve deeper into IPC in this context to better understand the disparity between research findings and real-world applications.

## Conclusion

Assessing and managing MCI can significantly benefit the control and mitigation of this condition. GPs are in a crucial position to identify associated risk factors, especially with the increasing occurrence of MCI in India. This study aimed to explore the knowledge, attitudes, and practices of Indian GPs regarding assessing and managing MCI. The findings indicate a limited understanding among GPs but also a significant potential for enhancing their knowledge about MCI. Additionally, fostering more positive attitudes toward managing the disorder and caring for patients and their families can greatly benefit overall care. Higher education and training seem to contribute to better knowledge and practice scores, respectively. However, a more comprehensive approach is necessary to address the growing incidence of MCI effectively. While GPs demonstrate good perception scores, actual involvement in IPC remains limited, highlighting the need for increased practical implementation and research in India. This underscores the importance of not only providing training on MCI but also promoting an IPC approach that effectively addresses the condition in its early stages. Such an approach requires collaboration among doctors, family members, and allied health professionals, facilitated by a structured platform for effective interaction. This strategy will not only enhance patient care but also ensure optimal utilization of medical resources and the workforce.

## Electronic supplementary material

Below is the link to the electronic supplementary material.

Supplementary Material 1

Supplementary Material 2

## Data Availability

The datasets used and/or analysed during the current study are available from the corresponding author on reasonable request.

## Abbreviations

MCI: Mild Cognitive impairment
GPs: General Physicians
IPC: Interprofessional Collaborative
KAP: Knowledge, Attitude, Practice
MD: Doctor of Medicine
MBBS: Bachelor of Medicine and Bachelor of Surgery
DNB: Diploma of National Board
CVRFs: Cardiovascular Risk Factors
SLPs: Speech Language Pathologists
CBME: Curriculum-Based Medical Education
VCI: Vascular cognitive impairment
