# Supplementary material for: Knowledge, Attitudes, and Practices of General Physicians towards Mild Cognitive Impairment amidst an evolving era of Interprofessional Collaboration: Insights from a small-scale survey in India
Source: BMC Prim Care. 2025 Feb 19;26:46. doi: 10.1186/s12875-025-02748-7 (PMC11837318; doi:10.1186/s12875-025-02748-7)
Supplement: Supplementary file 1 — Supplementary Material 1 [file 12875_2025_2748_MOESM1_ESM.docx]

**Modification in Questionnaire adopted from Lu et al. (2022)**

| Original Question | Revised Question |
| --- | --- |
| Sociodemographic data | |
| Please make sure whether you`d like to take this survey | Do you wish to participate in the survey |
| Gender | Gender of participant |
| Age | Age (in years) of participant |
| Education Level | Education Level of participant |
| Working department | Working setup |
| Length of GP Experience | Clinical experience (in years) after completion of MBBS |
| Additional Question: Location of practice (City and State) | |
| MCI detection and management context experience | |
| Daily visiting patients | Average number of patients that you treat on a daily basis |
| MCI training | Have you been trained to detect and manage individuals with MCI? |
| Proportion of patients with memory disorder last month | Proportion of patients with memory related issues seen by you last month |
| Proportion of patients with psychiatric symptoms last month | Proportion of patients with cardiovascular risk factors (Diabetes, Hypertension, Dyslipidaemia, Obesity) seen by you last month |
| MCI detection and management experience | Do you have experience in detecting and managing individuals with MCI? |
| Additional Question: Which among the following do you consider to be necessary while diagnosing MCI? | |
| Knowledge of clinicians/doctors toward MCI detection and management | |
| What is the estimated percentage of people over 60 years old who have MCI in community setting? | What is the estimated percentage of people with Cardiovascular Risk Factors who have MCI in the community setting? |
| Which are modifiable risk factors affecting cognitive function? | Which among the following are risk factors affecting cognitive functions? |
| Which of the following is NOT required to diagnose MCI according to Peterson diagnosis criteria? | Which among the following are the criteria used for diagnosis of Mild Cognitive Impairment?  (More than one answers can be selected) |
| Which of the following doctors are necessary in MCI diagnosis? | Which of the following healthcare professionals are necessary in MCI diagnosis? |
| Which one of the following is the most commonly used MCI screening scale? | Which one of the following is the most commonly used MCI screening scale? |
| Which statement is false concerning treatment of MCI? | Which statement is false concerning treatment of MCI? |
| What is the average progression rate after onset of MCI? | The proportion of people with MCI who progress to Dementia. |
| What are effective non-pharmacological MCI interventions? | Which among the following are effective non-pharmacological MCI interventions? |
| Attitudes of clinicians/doctors towards MCI detection and Management | |
| MCI is not a disease, but a degenerative aging process. | MCI is not a disease, but a degenerative aging process. |
| There are more advantages than disadvantages to finding out if someone has MCI. | There are more advantages than disadvantages to finding out if someone has MCI. |
| All patients suspected of MCI should undergo a diagnostic evaluation. | All patients suspected of MCI should undergo a diagnostic evaluation. |
| Early recognition and management can delay the progression to Alzheimer`s disease | Early recognition and management of MCI can delay the progression to Dementia. |
| There are more advantages than disadvantages to manage MCI patients with medicine | There are more advantages than disadvantages in managing individuals with MCI using pharmacological methods |
| There are more advantages than disadvantages to manage MCI patients with non-pharmaceutical methods. | There are more advantages than disadvantages in managing individuals with MCI using non-pharmacological methods |
| Patients with dementia can be a drain on medical and social resources | Patients with dementia can be a drain on medical and social resources. |
| Disclosure of disease could cause stress and frustration to patients and their families | Disclosure of Mild Cognitive Impairment could cause stress and frustration to the patients. |
| Disclosure of disease could cause embarrassment or discomfort for doctors | Disclosure of MCI to the patients could cause embarrassment or discomfort for doctors. |
| Being diagnosed with MCI could provide some hope for patients compared with being diagnosed with Alzheimer’s` disease. | Being diagnosed with MCI could provide some hope for patients compared to being diagnosed with Dementia. |
| MCI detection and management provide no economic benefits. | MCI detection and management provides no economic benefits to the society. |
| It`s GPs responsibility to recognise MCI in the primary care setting. | It`s responsibility of the General practitioner to recognize MCI in the primary care setting. |
| It`s GPs responsibility to managing MCI in the primary care setting | It`s responsibility of the General practitioner to manage MCI in the primary care setting. |
| Additional Question: In the diagnosis and management of MCI, an Interprofessional Collaborative approach would be highly effective | |
| Practice of Clinicians/Doctors toward MCI detection and Management | |
| Taking memory disorder as the criteria for MCI detection | I take symptoms of memory loss as the criteria for Mild Cognitive Impairment detection. |
| Taking psychiatric symptoms as the criteria for MCI detection | I take psychiatric symptoms as the criteria for Mild Cognitive Impairment detection |
| I would ask if a patient has Alzheimer’s` disease family history | I generally ask if a patient has family history of Dementia. |
| I would detect MCI risk factors | I usually screen for Mild Cognitive Impairment in individuals with cardiovascular risk factors (Diabetes, Hypertension, Dyslipidemia, Obesity, Smoking) |
| I would utilise the MCI screening methods | I would utilize a screening scale for detection of Mild Cognitive Impairment. |
| I would get specialist advice for final diagnosis by transference | I usually refer a suspected individual with Mild Cognitive Impairment to a specialist for final diagnosis. |
| I would discuss the probable diagnosis with the patient. | I usually discuss the probable diagnosis of Mild Cognitive Impairment with the patient |
| I would discuss the probable diagnosis with the family. | I would discuss the probable diagnosis of MCI with the patient’s family. |
| I would prescribe medications | I usually prescribe medications for treatment of Mild Cognitive Impairment. |
| I would provide non-pharmacological interventions. | I usually provide non-pharmacological interventions for treatment of Mild Cognitive Impairment. |
| Additional Questions:   1. I take an Interprofessional Collaborative (IPC) approach to diagnose Mild Cognitive Impairment. (ICP refers to multiple health professionals working together to provide coordinated and comprehensive care) 2. I take an Interprofessional Collaborative (IPC) approach to manage Mild Cognitive Impairment. (ICP refers to multiple health professionals working together to provide coordinated and comprehensive care) | |
