# Supplementary material for: Knowledge, Attitudes, and Practices of General Physicians towards Mild Cognitive Impairment amidst an evolving era of Interprofessional Collaboration: Insights from a small-scale survey in India
Source: BMC Prim Care. 2025 Feb 19;26:46. doi: 10.1186/s12875-025-02748-7 (PMC11837318; doi:10.1186/s12875-025-02748-7)
Supplement: Supplementary file 2 — Supplementary Material 2 [file 12875_2025_2748_MOESM2_ESM.doc]

Survey on the **Knowledge, Attitudes, and Practices of General Physicians Towards Mild Cognitive Impairment**

Dear Physicians,

Greetings! We are conducting a questionnaire survey on the preparedness of doctors in detecting and managing mild cognitive impairment (MCI) in India. This study is conducted in collaboration between the Department of General Medicine and the Department of Audiology and Speech language pathology, Kasturba Medical College, Mangalore, Manipal Academy of Higher Education, Manipal. This study has obtained required ethical clearance from the appropriate authorities, ensuring compliance with the ethical standards of the institute. If you have any questions or concerns, please reach out to AS [PhD Scholar, Department of Audiology and Speech language pathology, Kasturba Medical College] at XXXXXXXXX.

You are invited to complete this 10-minute questionnaire. Your responses will be of great value. We hope to learn from your experience and perceptions, which will help us determine how doctors can effectively detect and manage MCI in India. The survey is completely voluntary and anonymous. Your responses will be used only for academic research.

Do you wish to participate in this survey?

**☐ Yes**

**☐ No**

**Kindly fill in the following details about yourself**

1. **Gender of participant**

☐Male

☐Female

☐Others

1. **Age (in years) of participant**

☐ < 30
☐ 30-39
☐ 40-49
☐ ≥ 50

1. **Educational level of participant**

☐ MBBS

☐ MD/DNB General/Internal Medicine

☐ MD/DNB Family Medicine

☐ Others (Fellowships/ Super specializations): _________

1. **Working Setup**

☐ Private practice

☐ Medical College

☐ Government Service Clinic

☐ Corporate Hospital

☐ Others: _________________

1. **Location of practice (City and State)**

______________________________

1. **Clinical experience (in years) after completion of MBBS**

☐ < 5
☐ 5-9
☐ 10-14
☐ ≥15

**Experience in detection and management of Mild Cognitive Impairment**

1. **Average number of patients that you treat on a daily basis**

☐ < 20

☐ 20-50

☐ 50-99

☐ ≥100

1. **Have you been trained to detect and manage individuals with Mild Cognitive Impairment?**

☐ Yes

☐ No

☐ Unsure

1. **Proportion of patients with memory related issues seen by you last month**

☐ 0

☐ <10%

☐ 10-29%

☐ ≥ 30%

☐ Unsure

1. **Proportion of patients with cardiovascular risk factors (Diabetes, Hypertension, Dyslipidemia, Obesity, Smoking) seen by you last month**

☐ 0

☐ <10%

☐ 10-29%

☐ ≥ 30%

☐ Unsure

1. **Do you have experience in detecting and managing individuals with Mild Cognitive Impairment?**

☐ Yes

☐ No

☐ Unsure

**Knowledge of clinicians toward Mild Cognitive Impairment detection and management (Answer to the best of your knowledge)**

1. **What is the estimated percentage of people with Cardiovascular Risk Factors who have Mild Cognitive Impairment in the community setting?**

☐ <10%

☐ 10-29%

☐ ≥ 30%

☐ Unsure

*Max Attainable Score: 1 point*

1. **Which among the following do you think are risk factors contributing to a decline in cognitive functions? (More than one answers can be selected)**

☐ Hypertension

☐ Diabetes Mellitus

☐ Hyperlipidemia

☐ Family History

☐ Lack of Exercise

☐ Alcohol or drug abuse

☐ Smoking

☐ Stressful lifestyle

☐ Cognitive engagement

☐ Sleep Habits

☐ Hearing Loss

☐ Depression

☐ Education level

☐ Employment status

☐ Others: ________

☐ Unsure

*Max Attainable Score: 14 points*

1. **Which of the following healthcare professionals are necessary in diagnosis of Mild Cognitive Impairment? (More than one answers can be selected)**

☐ Physicians

☐ Speech Language Pathologist

☐ Psychologist

☐ Psychiatrist

☐ Neurologist

☐ All of the above

☐ None of the above

☐ Unsure

*Max Attainable Score: 6 points (If all the five professionals and/or ‘All of the above’ option is chosen then participant would get 6 points)*

1. **The proportion of people with Mild Cognitive Impairment who progress to Dementia.**

☐ <10%

☐ 10-19%

☐ 20-39%

☐ ≥40%

☐ Unsure

*Max Attainable Score: 1 point*

1. **Which among the following are the criteria used for diagnosis of Mild Cognitive Impairment? (More than one answers can be selected)**

☐ Peterson’s criteria

☐ DSM-V Criteria

☐ National Institute of Aging Alzheimer’s Association Criteria

☐ MCI: Manchester Approach

☐ Others: ___________________

☐ Unsure

*Max Attainable Score: 4 points*

1. **Which one of the following is the most commonly used MCI screening scale? (More than one answers can be selected)**

☐ The Montreal Cognitive Assessment (MoCA)

☐ Mini-Mental State Examination (MMSE)

☐ Addenbrooke's Cognitive Examination-R (ACE-R)

☐ ICMR Neuro Cognitive Toolbox (ICMR-NCTB)

☐ Clinic Dementia Rating Score (CDR)

☐ Activities of Daily Living (ADL)

☐ General Practitioner assessment of cognition (GPCOG) scale

☐ Others: _______________

☐ Unsure

*Max Attainable Score: 7 points*

1. **Which statement is FALSE concerning treatment of MCI?**

☐ Management of MCI may reverse progression of cognitive symptoms

☐ It is important to rule out and treat reversible risks for MCI

☐ Treating the cardiovascular risk factors may reverse MCI

☐ The cognitive function may reverse automatically in some MCI cases

☐ Unsure

*Max Attainable Score: 1 point*

1. **Which among the following do you consider to be necessary while diagnosing MCI? (More than one answers can be selected)**

☐ Neuropsychological assessment

☐ Neuropsychiatric assessment

☐ Thyroid-stimulating hormone (TSH), triiodothyronine (T3), and free thyroxine (FT4)

☐ Vitamin B12 serum

☐ Serum Folic acid

☐ Venereal Disease Research Laboratory Test (VDRL)

☐ Computed tomography (CT) of the brain

☐ Magnetic resonance imaging (MRI) of the brain

☐ Single photon emission computed tomography (SPECT) scan of the brain

☐ HIV

☐ Others: _______

☐ Unsure

*Max Attainable Score: 10 points*

1. **Which among the following are effective non-pharmacological interventions for Mild Cognitive Impairment? (More than one answers can be selected)**

☐ Aerobic exercise

☐ Mediterranean diet

☐ Music

☐ Social activities (family gatherings, attending functions)

☐ Yoga

☐ Cognitive training

☐ Others: ­­­­­­­­­­________________________________

☐ Unsure

*Max Attainable Score: 6 points*

*Total Attainable score in Knowledge Section: 50 points*

**Attitudes of clinicians/doctors towards Mild Cognitive Impairment (MCI) detection and Management**

|  | **Strongly agree**  (5 points) | **Agree**  (4 points) | **Unsure**  (3 points) | **Disagree**  (2 points) | **Strongly disagree**  (1 point) |
| --- | --- | --- | --- | --- | --- |
| 1. MCI is not a disease, but a normal aging process. | ☐ | ☐ | ☐ | ☐ | ☐ |
| 1. There are more advantages than disadvantages to finding out if someone has MCI. | ☐ | ☐ | ☐ | ☐ | ☐ |
| 1. All patients suspected of MCI should undergo a diagnostic evaluation. | ☐ | ☐ | ☐ | ☐ | ☐ |
| 1. Early recognition and management of MCI can delay the progression to Dementia. | ☐ | ☐ | ☐ | ☐ | ☐ |
| 1. There are more advantages than disadvantages in managing individuals with MCI using pharmacological methods. | ☐ | ☐ | ☐ | ☐ | ☐ |
| 1. There are more advantages than disadvantages in managing individuals with MCI using non-pharmacological methods. | ☐ | ☐ | ☐ | ☐ | ☐ |
| 1. Patients with dementia can be a drain on medical and social resources. | ☐ | ☐ | ☐ | ☐ | ☐ |
| 1. Disclosure of MCI could cause stress and frustration to the patients. | ☐ | ☐ | ☐ | ☐ | ☐ |
| 1. Disclosure of MCI could cause stress and frustration to the families | ☐ | ☐ | ☐ | ☐ | ☐ |
| 1. Disclosure of MCI to the patients could cause embarrassment or discomfort to doctors. | ☐ | ☐ | ☐ | ☐ | ☐ |
| 1. Being diagnosed with MCI could provide some hope for patients compared to being diagnosed with Dementia. | ☐ | ☐ | ☐ | ☐ | ☐ |
| 1. MCI detection and management provides no economic benefits to the society. | ☐ | ☐ | ☐ | ☐ | ☐ |
| 1. It is responsibility of the doctor to recognize MCI in the primary care setting. | ☐ | ☐ | ☐ | ☐ | ☐ |
| 1. It is responsibility of the doctor to manage MCI in the primary care setting. | ☐ | ☐ | ☐ | ☐ | ☐ |
| 1. In the diagnosis and management of Mild Cognitive Impairment, an Interprofessional Collaborative (IPC) approach would be highly effective. (IPC refers to multiple health professionals working together to provide coordinated and comprehensive care) | ☐ | ☐ | ☐ | ☐ | ☐ |

*Total Attainable Score in Attitudes section: 75 points (Maximum 5 points for each question)*

*Reverse scoring applicable on questions: 1, 7, 8, 9, 10, 12*

**Practice of Clinicians/Doctors toward MCI detection and management**

|  | **Always**  (5 points**)** | **Usually** (4 points) | **Sometimes** (3 points) | **Seldom**  (2 points) | **Never**  (1 point) | **Not Applicable** |
| --- | --- | --- | --- | --- | --- | --- |
| 1. I take symptoms of memory loss as the criteria for MCI detection. | ☐ | ☐ | ☐ | ☐ | ☐ | ☐ |
| 1. I take psychiatric symptoms as the criteria for MCI detection | ☐ | ☐ | ☐ | ☐ | ☐ | ☐ |
| 1. I generally ask if a patient has family history of Dementia. | ☐ | ☐ | ☐ | ☐ | ☐ | ☐ |
| 1. I usually screen for MCI in individuals with cardiovascular risk factors (Diabetes, Hypertension, Dyslipidemia, Obesity, Smoking) | ☐ | ☐ | ☐ | ☐ | ☐ | ☐ |
| 1. I usually utilize a screening scale for detection of MCI. | ☐ | ☐ | ☐ | ☐ | ☐ | ☐ |
| 1. I usually refer a suspected individual with MCI to a specialist for final diagnosis. | ☐ | ☐ | ☐ | ☐ | ☐ | ☐ |
| 1. I usually discuss the probable diagnosis of MCI with the patient. | ☐ | ☐ | ☐ | ☐ | ☐ | ☐ |
| 1. I usually discuss the probable diagnosis of MCI with the patient’s family. | ☐ | ☐ | ☐ | ☐ | ☐ | ☐ |
| 1. I take an Interprofessional Collaborative approach to diagnose MCI. (IPC refers to multiple health professionals working together to provide coordinated and comprehensive care) | ☐ | ☐ | ☐ | ☐ | ☐ | ☐ |
| 1. I take an Interprofessional Collaborative approach to manage MCI | ☐ | ☐ | ☐ | ☐ | ☐ | ☐ |
| 1. I usually provide non-pharmacological interventions for treatment of MCI. | ☐ | ☐ | ☐ | ☐ | ☐ | ☐ |
| 1. I usually prescribe medications for treatment of MCI | ☐ | ☐ | ☐ | ☐ | ☐ | ☐ |

*Total Attainable Score in Practices section: 60 (Maximum 5 points for each question)*

*"Not Applicable" option, when selected, was scored as 1 point.*
